# Supplementary material for: Distinct Phenotypes of Kidney Transplant Recipients in the United States with Limited Functional Status as Identified through Machine Learning Consensus Clustering
Source: J Pers Med. 2022 May 25;12(6):859. doi: 10.3390/jpm12060859 (PMC9225038; doi:10.3390/jpm12060859)
Supplement: Supplementary file 1 [file jpm-12-00859-s001.zip › jpm-1719041-supplementary.pdf]

Supplementary Methods

Cluster derivation

We applied an unsupervised ML approach to develop clinical phenotypes of kidney transplant recipients with limited functional status in the UNOS/OPTN database by conducting unsupervised consensus clustering.[1] We performed consensus clustering analysis on the whole study population. We initially assessed the distribution and missingness in phenotyping variables. Subsequently, missing data were imputed through multiple imputation using multivariate imputation by chained equations (MICE),[2] and non-normal data were z-score normalized. Multiple imputation is a widely used approach to estimate variables when data are missing at random. MICE is optimal when less than 30% of a variable's data are missing.[3-8] All of the extracted variables in our study had missing data ≤5% (Table S2). We subsequently applied clustering using the consensus cluster algorithm. The algorithm begins by subsampling a proportion of items and a proportion of features from a data matrix. Each subsample is then partitioned into up to groups (k) by a user-specified clustering algorithm. This process is repeated for a specified number of times. Pairwise consensus values, defined as 'the proportion of clustering runs in which two items are grouped together', are calculated and stored in a consensus matrix (CM) for each cluster. Clustering settings used were as follows: maximum number of clusters, 10; number of iterations, 100; subsampling fraction, 0.8; clustering algorithm, K-means; Euclidean distance).[1] The number of potential clusters ranges from 2 to 10, to avoid producing an excessive number of clusters that would not be clinical useful. Pairwise consensus values, defined as 'the proportion of clustering runs in which two items are [grouped] together[1], are calculated and stored in a CM for each k. Then for each k, a final agglomerative hierarchical consensus clustering using distance of 1−consensus values is completed and pruned to k groups, which are called consensus clusters.

The clustering algorithm is to maximize the potential number of clusters while maintaining high cluster consensus. The optimal number of clusters was determined by examining the CM heat map, cumulative distribution function, cluster-consensus plots with the within-cluster consensus scores, and the proportion of ambiguously clustered pairs (PAC).[9, 10] The within-cluster consensus score, ranging between 0 and 1, is defined as the average consensus value for all pairs of individuals belonging to the same cluster.[10] A value closer to one indicates better cluster stability.[10] PAC, ranging between 0 and 1, is calculated as the proportion of all sample pairs with consensus values falling within the predetermined boundaries.[9] A value closer to zero indicates better cluster stability.[9] To examine the cluster profile, we calculated and graphically displayed the standardized mean differences of the variables between each cluster and the overall study population. Calculation of the standardized difference of each parameter used the cutoff of ±0.3 to show subgroup features with the key features for each cluster.

All cluster derivation analyses were performed using R, version 4.0.3 (RStudio, Inc., Boston, MA; http://www.rstudio.com/), with the packages of ConsensusClusterPlus (version 1.46.0)[10]. We imputed missing data through multivariable imputation by chained equation (MICE) method.[2] All analyses were two-tailed, and P value < .05 was considered statistically significant.

Table S1. Karnofsky Performance Score definitions[11]

	KPS	Definition
Normal	100	Normal; no complaints; no evidence of disease
	90	Able to carry on normal activity; minor signs or symptoms of disease
	80	Normal activity with effort; some sign or symptoms of disease
Capable of self-care	70	Cares for self; unable to carry on normal activity or do active work
Requires assistance	60	Requires occasional assistance, but is able to care for most personal needs
	50	Requires considerable assistance and frequent medical care
Disabled	40	Disabled; requires special care and assistance
	30	Severely disabled; hospitalization is indicated, although death not imminent
	20	Very sick; hospitalization necessary; active support treatment is necessary
	10	Moribund; fatal processes progressing rapidly
	0	Dead

Table S2 the number and percentages of missing data

	Missing data (total=3,205)
Recipient Age	0 (0)
Recipient male sex	0 (0)
ABO blood group	0 (0)
Body mass index	0 (0)
Kidney retransplant	0 (0)
Kidney donor status	0 (0)
Dialysis duration	29 (1)
Cause of end-stage kidney disease	0 (0)
Comorbidity	
- Diabetes mellitus	0 (0)
- Malignancy	0 (0)
- Peripheral vascular disease	59 (2)
PRA (%)	151 (5)
Positive HCV serostatus	0 (0)
Positive HBs antigen	0 (0)
Positive HIV serostatus	0 (0)
Functional status	0 (0)
Working income	123 (4)
Public insurance	0 (0)
US resident	0 (0)
Undergraduate education or above	70 (2)
Serum albumin	157 (5)
Donor age	0 (0)
Donor male sex	0 (0)
Donor race	0 (0)
History of hypertension in donor	0 (0)
KDPI	0 (0)
HLA mismatch	0 (0)
Cold ischemia time	34 (1)
Kidney on pump	0 (0)
Delay graft function	0 (0)
Allocation type	0 (0)
EBV status	145 (5)
CMV status	0 (0)
Induction immunosuppression	
- Thymoglobulin	0 (0)
- Alemtuzumab	0 (0)
- Basiliximab	0 (0)
- Other	0 (0)
- No induction	0 (0)
Maintenance Immunosuppression	
- Tacrolimus	0 (0)
- Cyclosporine	0 (0)
- Mycophenolate	0 (0)
- Azathioprine	0 (0)
- mTOR inhibitors	
- Steroid	0 (0)
	0 (0)

Table S3 proportion of clusters according to the regions

Region	N	Cluster 1	Cluster 2
1	83	60 (72)	23 (28)
2	575	382 (66)	193 (34)
3	218	156 (72)	62 (28)
4	306	216 (71)	90 (29)
5	430	299 (70)	131 (30)
6	13	10 (77)	3 (23)
7	630	404 (64)	226 (36)
8	280	214 (76)	66 (24)
9	135	102 (76)	33 (24)
10	413	296 (72)	117 (28)
11	122	77 (63)	45 (37)

Supplementary Figure S1. Consensus matrix heat map (k = 2) depicting consensus values on a white to blue color scale of each cluster

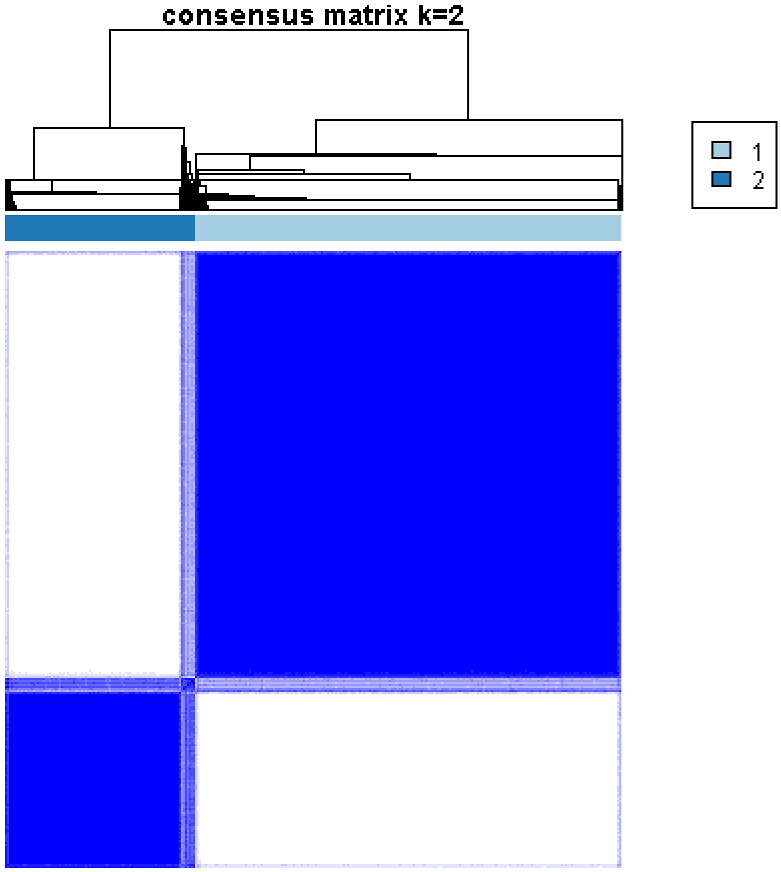

Supplementary Figure S2. Consensus matrix heat map (k = 3) depicting consensus values on a white to blue color scale of each cluster

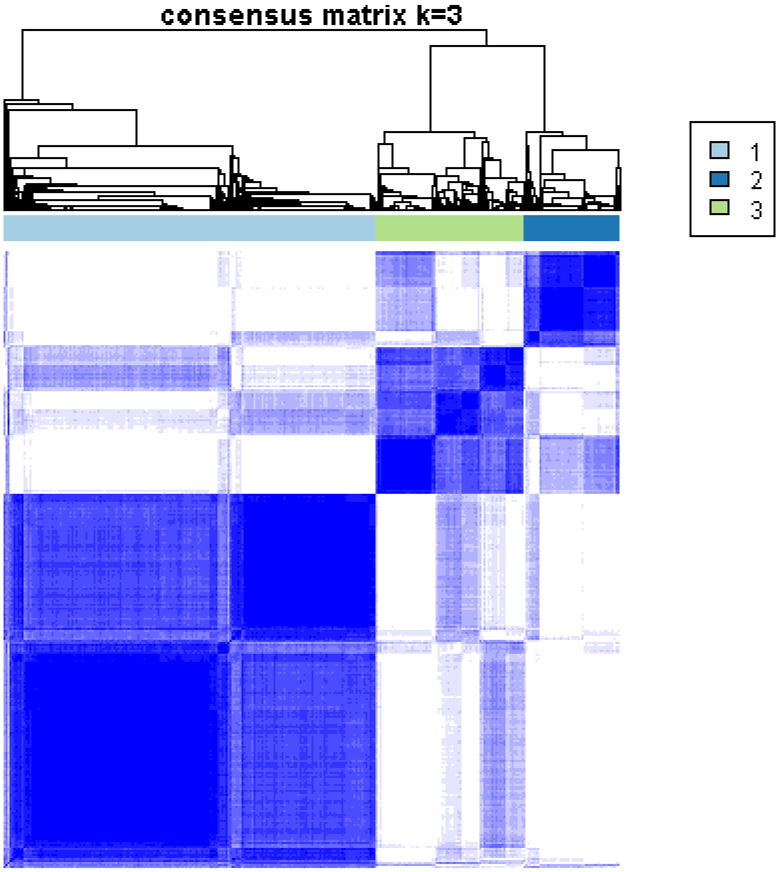

Supplementary Figure S3. Consensus matrix heat map (k = 4) depicting consensus values on a white to blue color scale of each cluster

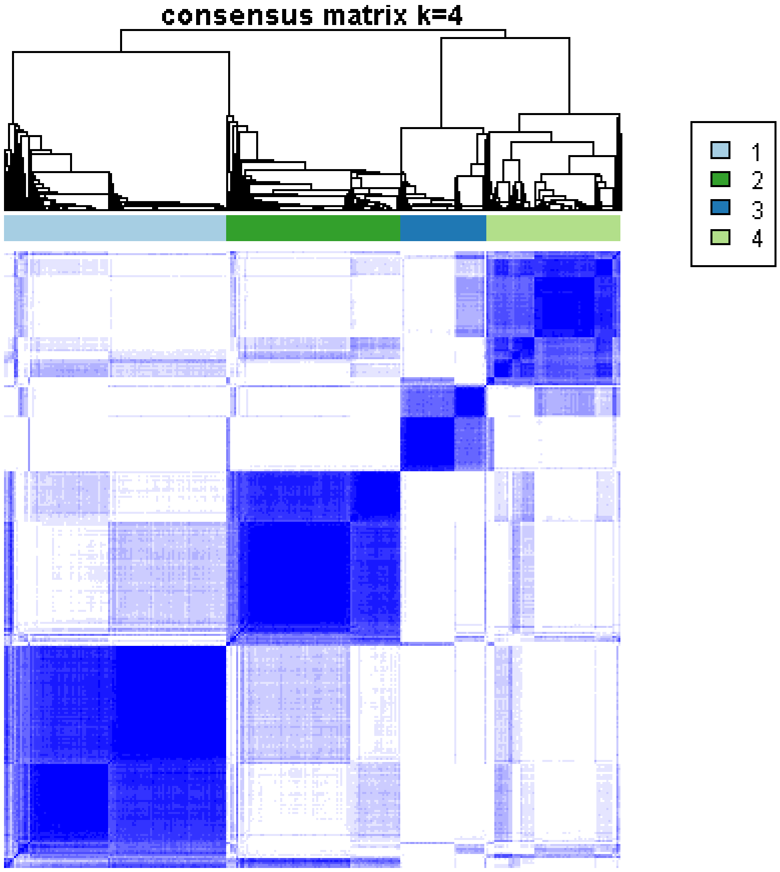

Supplementary Figure S4. Consensus matrix heat map (k = 5) depicting consensus values on a white to blue color scale of each cluster

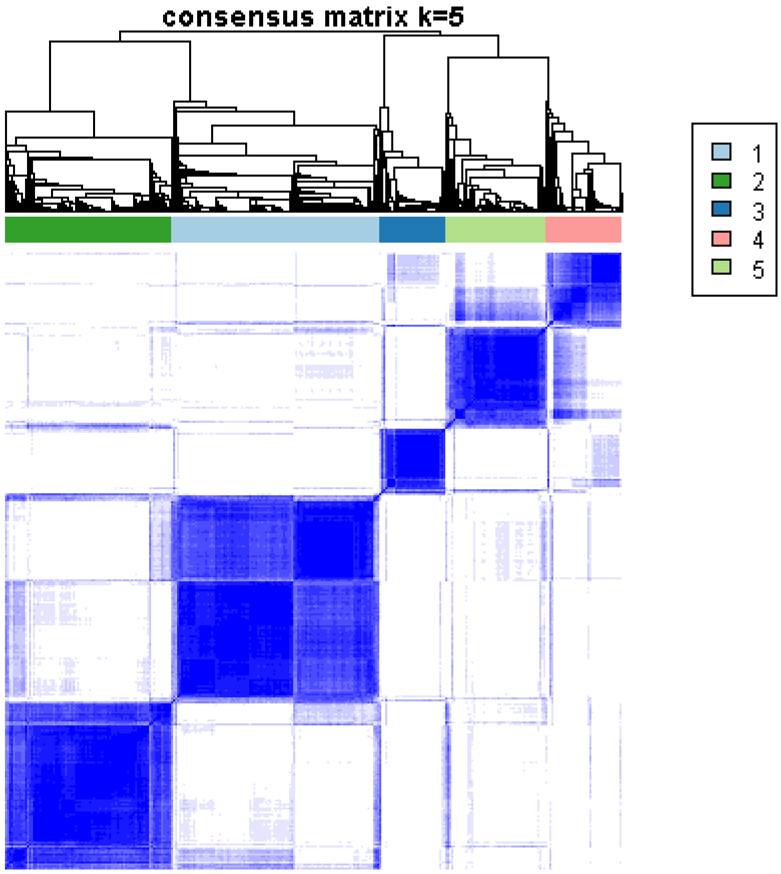

Supplementary Figure S5. Consensus matrix heat map (k = 6) depicting consensus values on a white to blue color scale of each cluster

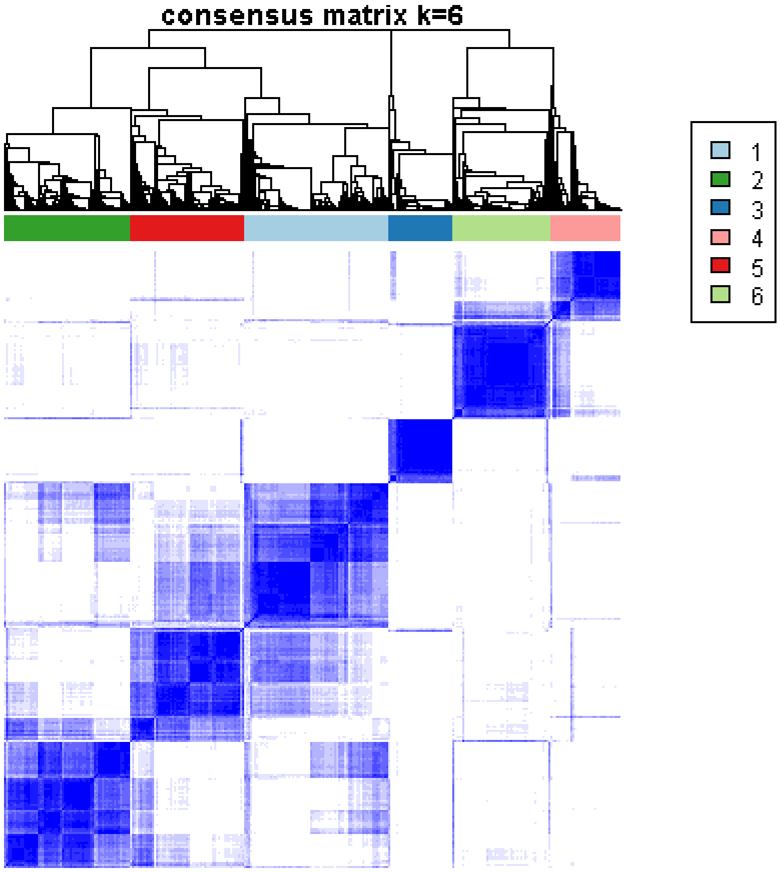

Supplementary Figure S6. Consensus matrix heat map (k = 7) depicting consensus values on a white to blue color scale of each cluster

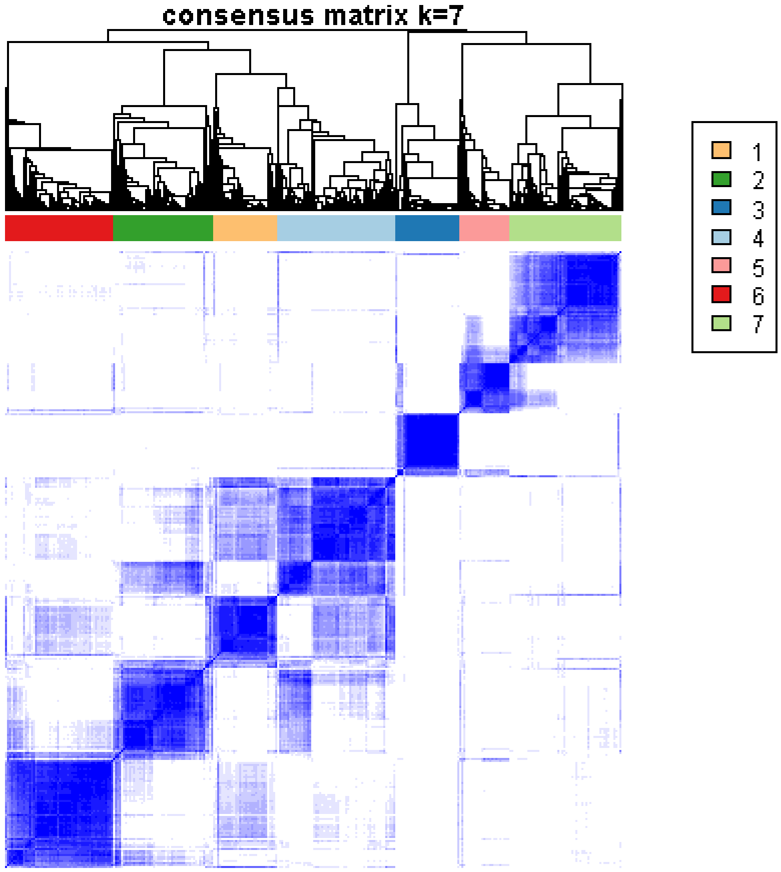

Supplementary Figure S7. Consensus matrix heat map (k = 8) depicting consensus values on a white to blue color scale of each cluster

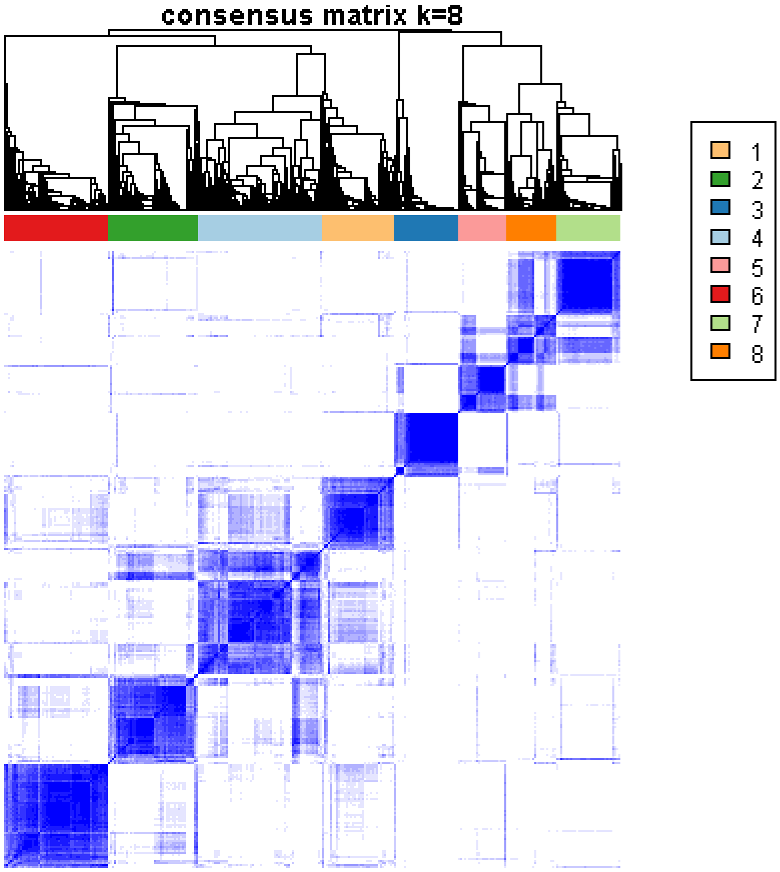

Supplementary Figure S8. Consensus matrix heat map (k = 9) depicting consensus values on a white to blue color scale of each cluster

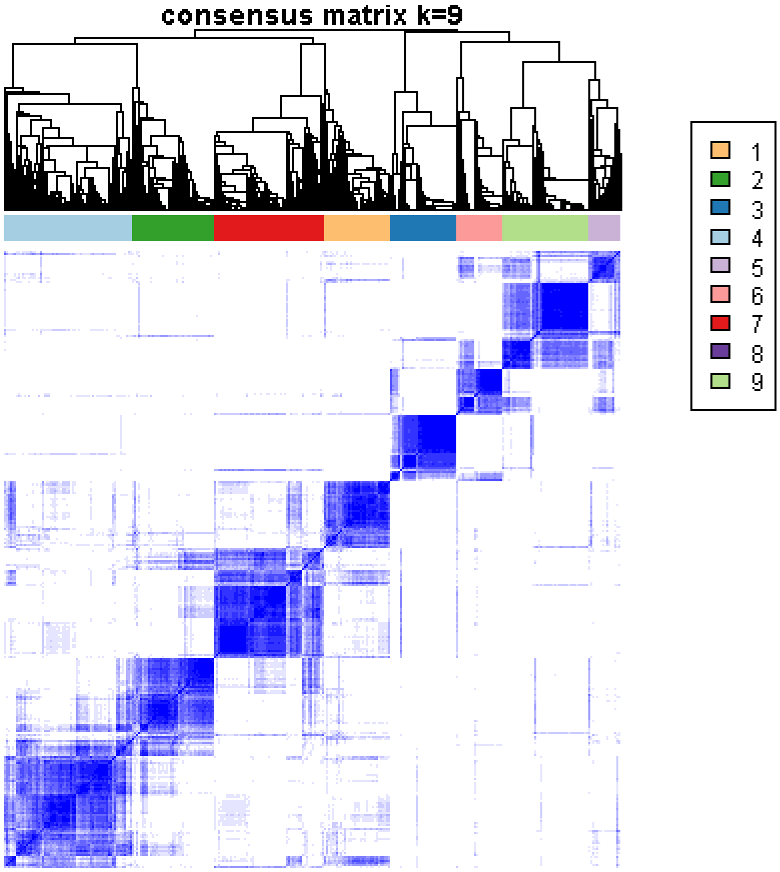

Supplementary Figure S9. Consensus matrix heat map (k = 10) depicting consensus values on a white to blue color scale of each cluster

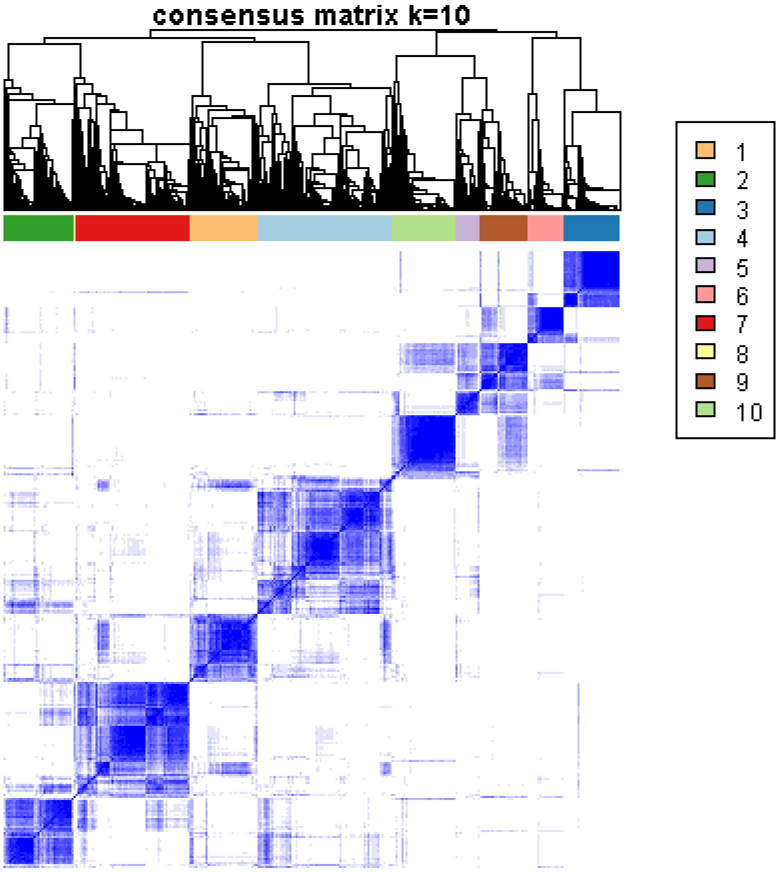

Supplementary Figure S10. A. Proportion of clusters according to the regions. B. OPTN regions.

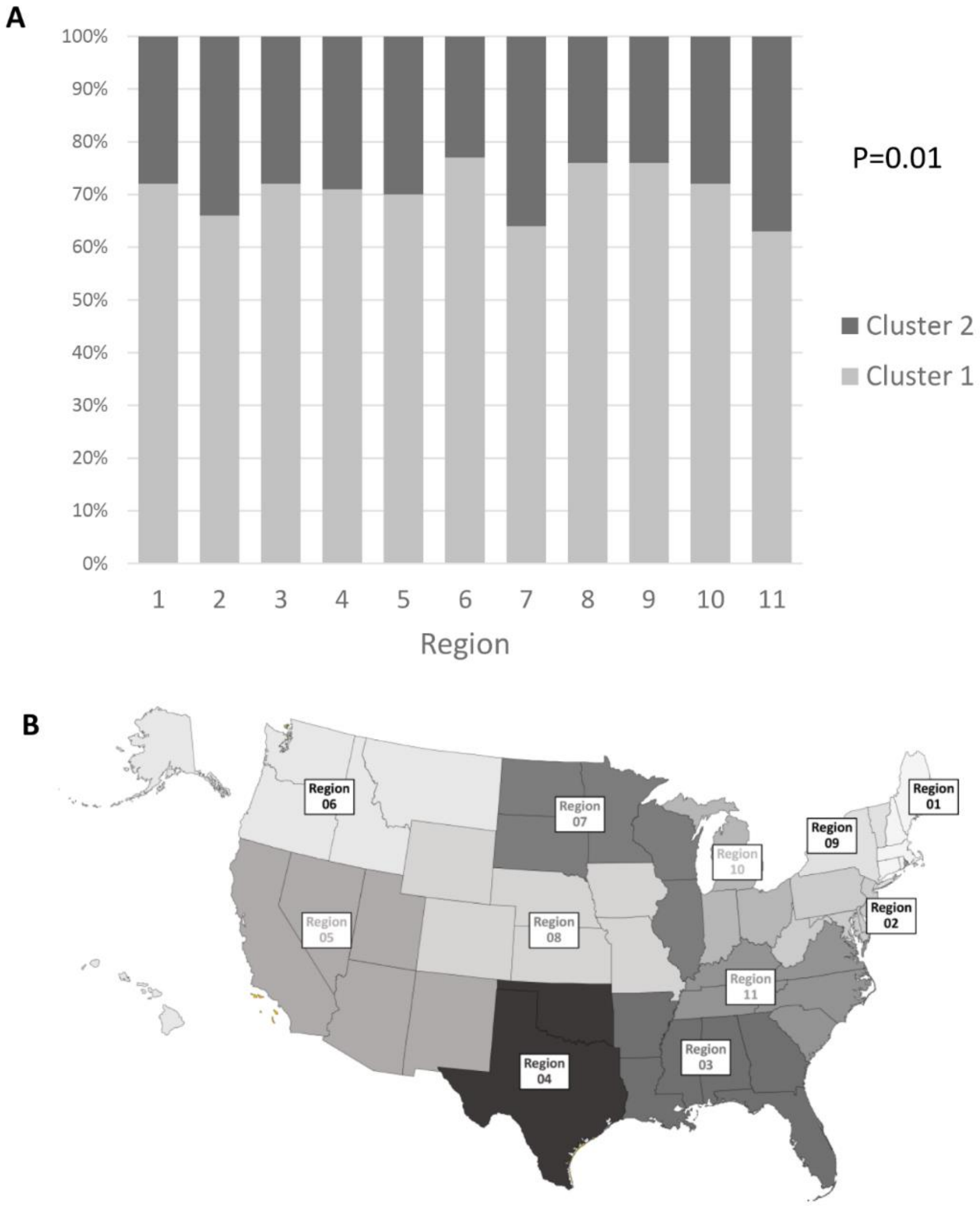

References

1. Monti S, Tamayo P, Mesirov J, Golub T: Consensus clustering: a resampling-based method for class discovery and visualization of gene expression microarray data. *Machine learning,* 52**:** 91-118, 2003
2. Van Buuren S, Groothuis-Oudshoorn K: mice: Multivariate imputation by chained equations in R. *Journal of statistical software,* 45**:** 1-67, 2011
3. Jannat-Khah DP, Unterbrink M, McNairy M, Pierre S, Fitzgerald DW, Pape J, Evans A: Treating loss-to-follow-up as a missing data problem: a case study using a longitudinal cohort of HIV-infected patients in Haiti. *BMC Public Health,* 18**:** 1269, 2018 10.1186/s12889-018-6115-0
4. Knol MJ, Janssen KJ, Donders AR, Egberts AC, Heerdink ER, Grobbee DE, Moons KG, Geerlings MI: Unpredictable bias when using the missing indicator method or complete case analysis for missing confounder values: an empirical example. *J Clin Epidemiol,* 63**:** 728-736, 2010 10.1016/j.jclinepi.2009.08.028
5. White IR, Carlin JB: Bias and efficiency of multiple imputation compared with complete-case analysis for missing covariate values. *Stat Med,* 29**:** 2920-2931, 2010 10.1002/sim.3944
6. White IR, Royston P, Wood AM: Multiple imputation using chained equations: Issues and guidance for practice. *Stat Med,* 30**:** 377-399, 2011 10.1002/sim.4067
7. Hedden SL, Woolson RF, Carter RE, Palesch Y, Upadhyaya HP, Malcolm RJ: The impact of loss to follow-up on hypothesis tests of the treatment effect for several statistical methods in substance abuse clinical trials. *J Subst Abuse Treat,* 37**:** 54-63, 2009 10.1016/j.jsat.2008.09.011
8. Donders ART, Van Der Heijden GJ, Stijnen T, Moons KG: A gentle introduction to imputation of missing values. *Journal of clinical epidemiology,* 59**:** 1087-1091, 2006
9. Șenbabaoğlu Y, Michailidis G, Li JZ: Critical limitations of consensus clustering in class discovery. *Sci Rep,* 4**:** 6207, 2014 10.1038/srep06207
10. Wilkerson MD, Hayes DN: ConsensusClusterPlus: a class discovery tool with confidence assessments and item tracking. *Bioinformatics,* 26**:** 1572-1573, 2010
11. O'Toole DM, Golden AM: Evaluating cancer patients for rehabilitation potential. *West J Med,* 155**:** 384-387, 1991
